# Peptide Folding and Binding Probed by Systematic Non-canonical Mutagenesis

**DOI:** 10.3389/fmolb.2020.00100

**Published:** 2020-06-24

**Authors:** Joseph M. Rogers

**Affiliations:** Department of Drug Design and Pharmacology, University of Copenhagen, Copenhagen, Denmark

**Keywords:** intrinsically disordered proteins (IDP), unnatural amino acids, cyclic peptides, genetic code reprogramming, deep mutational scanning

## Abstract

Many proteins and peptides fold upon binding another protein. Mutagenesis has proved an essential tool in the study of these multi-step molecular recognition processes. By comparing the biophysical behavior of carefully selected mutants, the concert of interactions and conformational changes that occur during folding and binding can be separated and assessed. Recently, this mutagenesis approach has been radically expanded by deep mutational scanning methods, which allow for many thousands of mutations to be examined in parallel. Furthermore, these high-throughput mutagenesis methods have been expanded to include mutations to non-canonical amino acids, returning peptide structure-activity relationships with unprecedented depth and detail. These developments are timely, as the insights they provide can guide the optimization of *de novo* cyclic peptides, a promising new modality for chemical probes and therapeutic agents.

## Introduction

Interactions between proteins are essential for the working of the cell (Rual et al., 2005). There is a great diversity in the structure and dynamics of protein binding. The classic case is where two folded protein domains dock, with minor conformational changes upon binding (Schreiber and Fersht, 1995; Jones and Thornton, 1996). At the other extreme, unfolded “intrinsically disordered proteins” (IDPs) can interact and remain dynamic and disordered even after binding (Mittag et al., 2008; Borgia et al., 2018; Schuler et al., 2019). Many protein interactions exist in-between these two extremes—a short peptide is disordered in isolation but folded when bound to a partner protein (Wright and Dyson, 2009; Yang et al., 2019). These peptide folding and binding reactions are widespread in biology (Tompa et al., 2014; Yan et al., 2016) and are especially enriched in eukaryotes and in proteins associated with disease (Uversky et al., 2008, 2014). We need a thorough understanding of how amino acid sequence affects the binding of these peptides. First, to rationalize their abundance and role in pathology. Second, to develop potent therapeutics able to mimic this mode of molecular recognition.

Folding upon binding reactions are necessarily multistep: Peptide and protein must diffuse into the same vicinity, the peptide must fold, interactions must form between peptide and partner protein, and the partner protein may change conformation—not necessarily in this order. Despite this complexity, many peptide interactions appear to be highly cooperative: At equilibrium, only two states are observed; the peptide is bound and folded, or unbound and disordered. Therefore, binding affinity can be captured by a single thermodynamic value, *K*_D_ or Δ*G*°. However, this strength of binding depends on numerous inter- and intra-chain interactions as well as the conformational preferences of the peptide and protein. Even if the three-dimensional structure of the bound state is known, it can be challenging to identify which chemical or sequence features truly drive binding. Mutagenesis can be used to disentangle these various contributions and explain how chemical structure leads to folding, binding and function. Here we describe the various approaches to mutagenesis, as applied to peptide folding and binding: traditional one-at-a-time alanine scanning, mutations to non-canonical amino acids, and state-of-the-art methods that allow huge numbers of mutations to be analyzed in parallel.

## Canonical One-at-a-Time Mutagenesis

A well-established method to probe protein-protein interactions is “alanine scanning.” In this, the bound structure is used to choose engaged side-chains for mutation to alanine. Of the canonical amino acids, mutation to alanine is preferred, as this is usually the most conservative chemical change, only removing interactions and not creating new ones. Site-directed mutagenesis and recombinant protein expression allow for relatively straightforward synthesis of mutant and wild-type peptides, which are then subjected to biophysical analysis to measure Δ*G*° and calculate ΔΔ*G* (here, $\Delta G_{\text{mut}}^{{}^{\circ}}-\Delta G_{\text{wt}}^{{}^{\circ}}$; positive when destabilizing).

**Figure 1:**
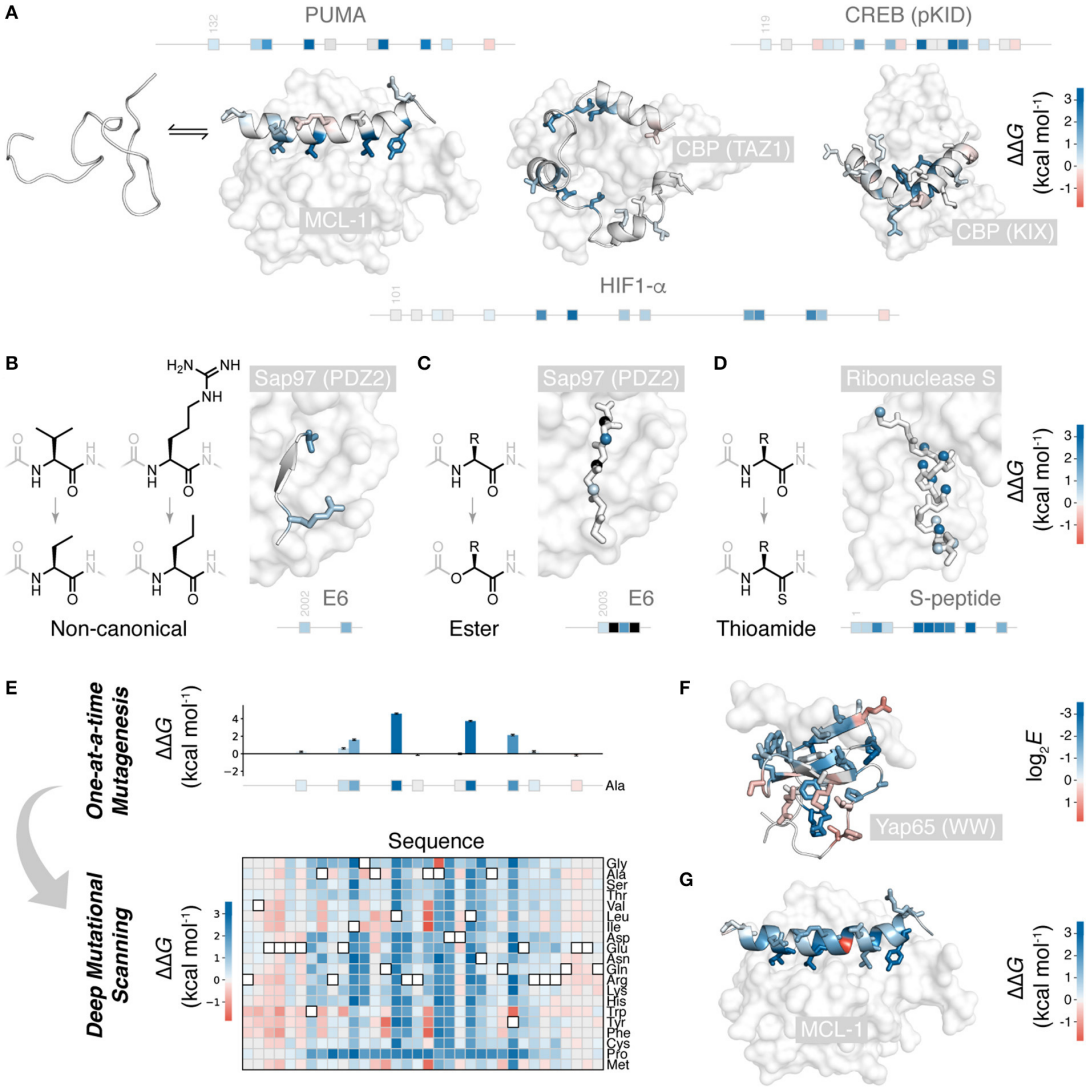
Mutational scanning to understand peptide folding and binding. **(A)** Many natural polypeptides are disordered in isolation, folding upon binding their partner protein. The IDP peptides PUMA (left) (Rogers et al., 2014; Crabtree et al., 2018), HIF-1α (center) (Lindstrom et al., 2018), and pKID of CREB (right) (Dahal et al., 2017) all fold to α-helices upon binding their protein partners. Side-chains mutated one-at-a-time to alanine shown, colored according to ΔΔ*G* (PDB 2ROC, 1L8C, and 1KDX). Position of these mutations on the primary structure of these peptides shown as boxes on a line. **(B)** Non-canonical side-chain mutagenesis applied to HPV18 E6 peptide folding to a β-strand upon interaction with a PDZ domain of Sap97 (Haq et al., 2012) (PDB 2I0L), colored according to ΔΔ*G* using **(A)** scale. **(C)** Non-canonical backbone mutagenesis, amide to ester, for the E6 peptide. Mutated amide nitrogen shown as spheres colored according to ΔΔ*G* using the **(A)** scale (Pedersen et al., 2014a). Black indicates those which could not be measured. **(D)** Backbone mutations to thioamide, applied to S-peptide binding S-protein (Bachmann et al., 2011) (PDB 2RLN). Mutated amide oxygens shown as spheres and colored according to ΔΔ*G*. **(E)** Deep mutagenesis scanning (DMS) allows thousands of mutations to be collected, and saturation mutagenesis to be performed. Shown is the saturation mutagenesis data for PUMA binding MCL-1 (Rogers et al., 2018). **(F)** Slice of DMS data for the folded YAP65 WW domain binding its peptide ligand; Ala mutations colored according to DMS enrichment score where negative score indicates weaker binding (Fowler et al., 2010) (PDB 1JMQ). **(G)** Slice of DMS data for PUMA binding MCL-1: all side-chains colored according to ΔΔ*G* for mutation to glycine, showing the one unexpected highly stabilizing mutation (Rogers et al., 2018).

Numerous peptide folding and binding systems have now been subjected to alanine scanning (Yang et al., 2019). Figure 1A shows the binding of three IDPs folding upon binding their partner proteins: PUMA (Rogers et al., 2014; Crabtree et al., 2018), HIF-1α (Lindstrom et al., 2018), and the pKID motif of CREB (Dahal et al., 2017) are all disordered in isolation, but fold to α-helices upon binding. Of the mutations studied, most are weakly destabilizing, apart from a small number of highly destabilizing mutations to key hydrophobic amino acids buried in the interaction. Hydrophobic to alanine mutations can destabilize up to 5 kcal mol^−1^, i.e., an ~5,000 × increase in *K*_D_. Agreeably, similar values of ΔΔ*G* are observed for comparable mutants in classical protein folding (Matouschek et al., 1989; Bava et al., 2004) and for interactions between folded proteins (Clackson and Wells, 1995; Schreiber and Fersht, 1995).

Backbone interactions are also an important component of folding and binding, as these govern chain dynamics, secondary structure formation, and, occasionally, direct backbone H-bonding with the partner protein. However, these interactions are challenging to study using canonical mutations. Only glycine and proline alter the peptide backbone. Proline mutations are structurally non-conservative due to the cyclic, N-alkyl structure of proline, and are, therefore, challenging to interpret. Glycine mutations are more useful. Glycine, with its larger range of accessible torsional angles, energetically favors unfolded states, and its lack of β-carbon causes a loss in hydrophobic packing. Thus, alanine to glycine mutations at solvent exposed positions can specifically destabilize α-helical folding and serve as a probe for this secondary structure formation (Serrano et al., 1992; Scott et al., 2007).

However, as a tool to study molecular interactions, these mutagenesis approaches suffer from being limited to the chemical structures of the 20 canonical amino acids. Higher-resolution structure-activity relationships are possible with access to non-canonical amino acids.

## Non-Canonical One-at-a-Time Mutagenesis

Non-canonical mutagenesis allows for high-resolution dissection of peptide chemical structure and its effect on peptide folding and binding. There are thousands of alternative, synthetically accessible non-canonical amino acids, and solid phase peptide synthesis (SPPS) allows these to be easily included in short peptides. With access to non-canonical amino acids, many more conservative side-chain mutations are possible. This is useful when mutation to alanine would be too destabilizing for the method in question or the role of a particular functional group or aliphatic carbon is to be studied. For example, in a study of the papilloma virus E6 peptide binding to a PDZ domain, the 3-carbon side-chain valine was mutated to the non-canonical 2-carbon aminobutyric acid, an energetically and structurally subtler modification than to alanine (Figure 1B) (Haq et al., 2012). Non-canonical mutagenesis is particularly valuable when the wild-type side-chain has multiple physicochemical characteristics. For example, the same E6 peptide has an arginine at the interface with its partner, and arginine has a hydrophobic side-chain topped by a guanidine head group, potentially forming hydrophobic and electrostatic/π-π stacking interactions, respectively, both of which would have been removed upon mutation to alanine. However, mutation to the non-canonical norvaline was able to assess the loss of the head-group only: a modest 0.8 kcal mol^−1^ destabilization (Haq et al., 2012) (Figure 1B).

Non-canonical mutagenesis also provides many opportunities to alter the peptide backbone: changing its H-bonding, conformational, and secondary structure propensities. For example, amide to ester mutations have been employed to probe peptide-protein interactions (Eildal et al., 2013; Pedersen et al., 2014b; Sereikaite et al., 2018). Mutation to ester replaces the amide H-bond donor with an acceptor and can therefore identify critical amide N-H interactions that drive folding and binding. Such mutations are particularly useful when the peptide of interest folds to a β-strand upon binding, forming multiple backbone H-bonds with the protein partner by adding to an existing β-sheet, as is the case the E6 peptide PDZ interaction (Figure 1C) (Eildal et al., 2013; Pedersen et al., 2014b). For the E6 peptide, mutations to ester were strongly destabilizing, preventing measurement of *K*_D_ when the replaced N-H was involved in β-sheet formation (Figure 1C). However, ester mutations also weaken the carbonyl H-bond acceptor and increase the conformational flexibility of the chain, explaining why even solved exposed amide N-H showed significant destabilization upon amide-to-ester substitution.

An isoelectronic backbone modification is amide to thioamide, swapping the amide oxygen for the sulfur (Figure 1D). Swapping amides for thioamides can be destabilizing, because of the larger sulfur, thioamides have a slightly restricted conformational space, the thiocarbonyl is a weaker H-bond acceptor and the thioamide N-H is a stronger H-bond donor (Walters et al., 2017). Applied to peptide folding and binding, these thioamide mutations have served as useful probes of secondary structure folding. Thioamide mutations have been tested in the S-peptide, which binds to a cleaved S-ribonuclease partner and folds to an α-helix upon binding (Figure 1D). Mutating amides with solvent-exposed carbonyls had little effect, whereas mutating those involved in helix formation significantly destabilized the complex. Interestingly, mutating amides involved in inter-chain H-bonding produced a similar destabilization to those involved in intra-chain helix formation (Bachmann et al., 2011).

However, these studies, much like one-at-a-time canonical mutagenesis, suffer from time-intensive peptide synthesis and purification, followed by low-throughput biophysical data collection. The need to individually synthesize and characterize each mutant means that a complete scan of the peptide or more than one mutation per site is generally not feasible. Figures 1A–D shows typical coverage of these one-at-a-time mutational scans, representing many months, even years, of work. One-at-a-time mutational studies are therefore incomplete, and important sequence or chemical features could be overlooked. Mutational scans with greater depth and coverage require a wholly different experimental approach.

## Deep Mutational Scanning

Over the last decade, deep mutational scanning (DMS) methods have emerged and have made it possible to study orders of magnitude more mutations than one-at-a-time approaches (Fowler and Fields, 2014). DMS allows thousands of mutants, up to hundreds of thousands, to be analyzed in parallel, in a single experiment. The throughput of DMS allows for saturation mutagenesis of peptides, i.e., the testing of all canonical mutations at all positions in the sequence (Figure 1E).

DMS methodology can be broken down in to four steps: (i) construction of a mutant DNA library; (ii) translation to proteins, retaining a link with the encoding DNA, e.g., by mRNA, ribosome, phage or yeast display, or using cell-based assays to maintain a link between phenotype and genotype; (iii) sorting of pooled libraries for function, e.g., pulldown or FACS for target binding, or cell survival; (iv) next-generation sequencing (NGS) to count variant populations, before and after sorting. The enrichment (or lack of) due to sorting, with normalization for the wild-type enrichment, can generate a score for each mutant that reports on its function or binding (Fowler and Fields, 2014). The raw enrichment scores contain significant information on relative binding affinities, the identity of beneficial and detrimental mutations, and their rank order. However, for carefully conducted experiments, these enrichment scores can be related to real thermodynamic values (*K*_D_ or Δ*G*°), either by assuming the function that relates the two (Weiss et al., 2000; Olson et al., 2014) or by empirically determining a calibration curve using a test set of mutants with known binding affinities (Rogers et al., 2018). Indeed, it is important to compare raw enrichment scores with a test set of mutants with known affinities, to validate that enrichment scores do, in fact, report on function.

The first systems examined using DMS were peptide folding upon binding reactions (Fowler et al., 2010; McLaughlin et al., 2012), except the folded protein partner was mutated rather than the peptide. Fowler et al. made over 600,000 variants of a WW domain and scored these for their ability to bind a short, proline-rich peptide (Fowler et al., 2010) (Figure 1F). As might be expected, mutations at the interface weakened the interaction. However, mutations distant from the interface, in the core of the WW domain, also lowered affinity. Likely, the WW domain can be destabilized and unfolded by mutation, to the point where even binding to the peptide cannot restore folding. Indeed, even though function (binding to peptide) is being assessed, careful analysis of double mutants can quantify the effect mutations have on folding (Araya et al., 2012; Olson et al., 2014). Many mutations appear to have non-additive effects in the double mutant libraries. However, this can be rationalized as approximately additive effects on folding stability; changes to folding stability will only affect function if the protein is destabilized enough to cross an important stability threshold, where even binding to the peptide cannot induce folding. This analysis can quantify the destabilization effect of mutations and even identify mutations that stabilize the folded structure (Araya et al., 2012).

DMS can also be used to analyze peptides which undergo folding and binding. Recently, we examined the peptide PUMA, which is intrinsically disordered in isolation, but folds to a long α-helix upon binding its partner proteins (Rogers et al., 2014; Crabtree et al., 2018) (Figure 1A). We conducted saturation mutagenesis of PUMA using the DMS method, using mRNA display to link each peptide with its encoding mRNA (Rogers et al., 2018) (Figure 1E). For a set of PUMA mutants with known *K*_D_ (Rogers et al., 2014), raw DMS enrichment scores correlated with binding affinity, and this correlation used to calibrate the DMS data and estimate ΔΔ*G* for all mutants. Mutations that stabilized the interaction were rare, but present, suggesting PUMA has not evolved for maximal affinity. Surprisingly, one of these stabilizing mutations was to glycine, which, as discussed above, usually destabilizes helical structure (Figure 1G), an anomaly unlikely to have been discovered by one-at-a-time mutagenesis.

DMS data enables new types of analysis. For example, general conclusions about protein mutagenesis can be reached, such as the finding that, on average, methionine is the most tolerated of the canonical amino acids (Gray et al., 2017). Perhaps the flexible, linear side-chain of methionine allows it to adapt to different structural contexts, or its moderate hydrophobicity (Moon and Fleming, 2011) is tolerated either buried or solvent exposed. Recently, DMS has been used to predict protein structures (Rollins et al., 2019; Schmiedel and Lehner, 2019), using the non-additivity of double mutants to identify residue pairs in contact, an approach which has the potential to generate structures for proteins resistant to current structural biology techniques. Perhaps one of the most promising uses of DMS is in the prediction of pathogenic mutations and to help understand the large numbers of genetic variants of unknown significance (Stein et al., 2019).

## Non-Canonical Deep Mutational Scanning

There are now a range of chemical synthetic tools to synthesize and study large collections of peptide mutants. These methods have great flexibility because they can include any of the many commercially available or synthetically accessible non-canonical amino acids. Examples include high-throughput SPPS (Simon et al., 2016), one-bead-one-compound libraries (Rezaei Araghi et al., 2016), peptide arrays (Lyamichev et al., 2017), and chemically synthesized DNA-encoded libraries (Denton et al., 2018; Wang et al., 2020). The latter have been used for saturation mutagenesis, creating highly detailed structure-activity maps for a peptide and its interaction with a target protein, described as “on-DNA Med Chem” (Wang et al., 2020).

Non-canonical peptide mutants can also be made by reprogramming the natural peptide synthesis machine: the ribosome. A highly adaptable method is the flexizyme system, which uses an artificial ribozyme to load non-canonical amino acids onto tRNAs for use during *in vitro* translation (Murakami et al., 2006). The number of non-canonical amino acids known to be accepted by the ribosome is in the hundreds, and there is a wide diversity of accepted chemical structures (Rogers and Suga, 2015).

**Figure 2:**
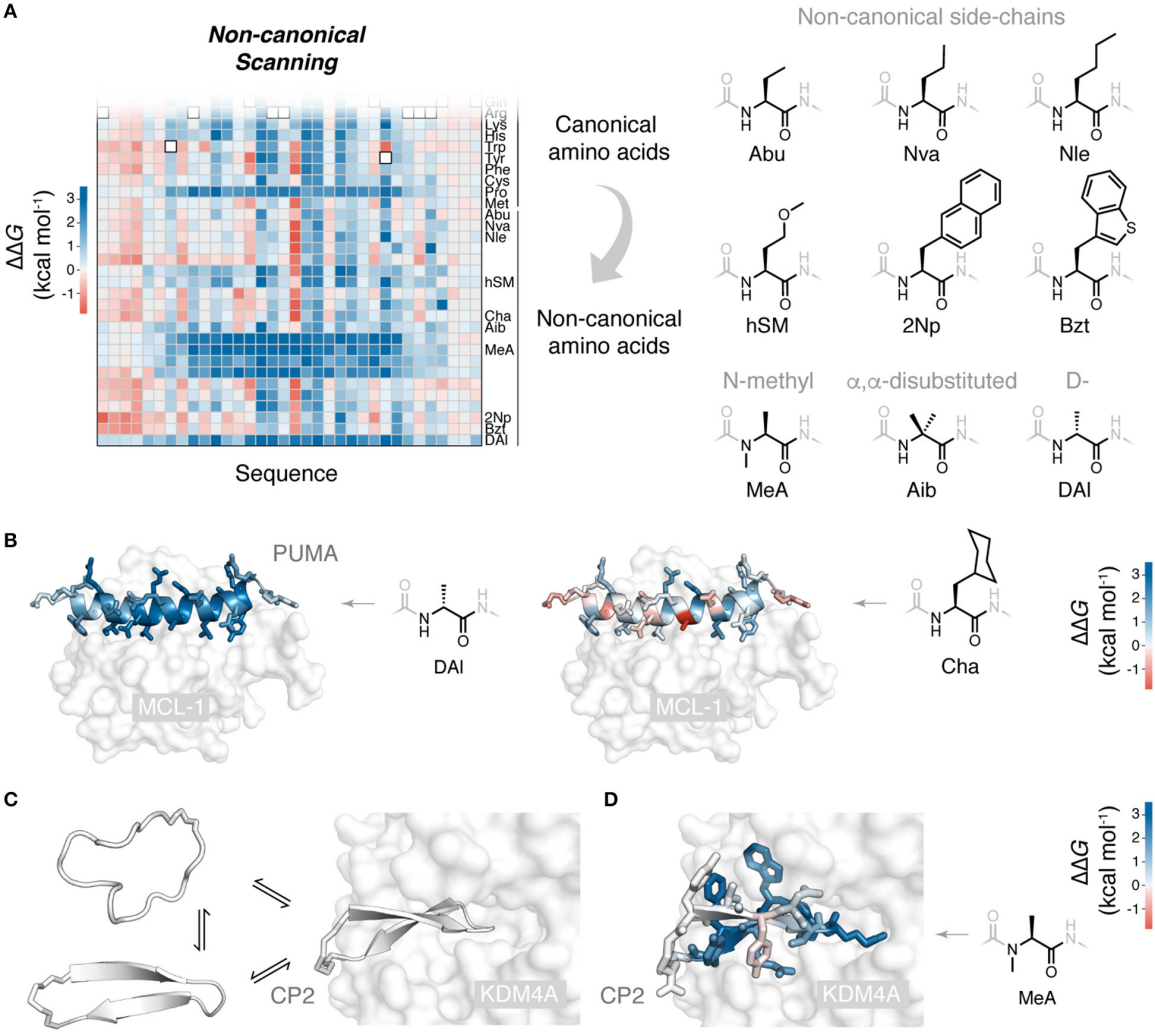
Non-canonical scanning and its application to drug-like cyclic peptides. **(A)** Reprogramming of ribosomal peptide synthesis allows deep mutational scanning to been expanded to include mutations to non-canonical amino acids. Non-canonical scanning (NCS) data for PUMA shown, with a selection of the non-canonical amino acids tested (Rogers et al., 2018). **(B)** Slices of NCS data for PUMA binding and scans with D-stereochemistry alanine (DAl) and cyclohexyl-alanine (Cha), side-chains colored according to ΔΔ*G*. **(C)** Cyclic peptides are a promising new modality due to their impressive protein binding abilities. Some degree of folding upon binding is expected for these binding reactions. Shown is the peptide CP2 binding to its target KDM4A (Kawamura et al., 2017) (PDB 5LY1). **(D)** NCS applied to the *de novo* cyclic peptide CP2 and its interaction with the histone demethylase KDM4A (Rogers et al., 2018): all CP2 side-chains colored according to ΔΔ*G* for mutation to non-canonical N-methyl alanine, identifying positions where backbone modifications can be made, to possibly increase protease resistance and membrane permeability (Rogers et al., 2018).

Powerfully, ribosomal synthesis of non-canonical mutants can be combined with deep mutational scanning analysis: non-canonical scanning (NCS). We recently applied NCS to the peptide PUMA, using the flexizyme system to build a saturation mutagenesis library including mutations to 21 diverse non-canonical amino acids, alongside the 20 canonical (Rogers et al., 2018). Mutations to multiple non-canonical aliphatic and aromatic side-chains were tested, as well as N-methylated, α,α-disubstituted and D-stereochemistry backbone altering amino acids (Figure 2A). Sorting of this library for binding to the partner protein, correcting for any differences in translation efficiency of the non-canonical amino acids (Rogers et al., 2018), generated a structure-activity map with unprecedented detail. For example, using a series of aliphatic non-canonical mutations, the progressive addition or removal of single aliphatic carbons could be assessed for every side-chain in the PUMA peptide. Interestingly, mutations to D-stereochemistry alanine were destabilizing across the α-helical region, and some substitutions to large aliphatic side-chain, such as cyclohexyl-alanine, could increase binding affinity (Figure 2B).

Systematic mutation methods such as NCS allow the chemical structures of biological peptides and their effect on folding and binding to be probed in great detail. However, these methods have utility in another sphere, the study of artificial binding peptides such as *de novo* cyclic peptides.

## Cyclic Peptides

Macrocyclic peptides are a promising modality in the search for new drugs and chemical probes (Vinogradov et al., 2019). Peptides with a cyclic topology can be protease resistant and membrane permeable and have potent, highly selective protein-binding abilities (Yudin, 2015; Naylor et al., 2017). Moreover, there are now efficient methods to screen large libraries of cyclic peptides to find *de novo* binding sequences (Obexer et al., 2017). Promisingly, these *de novo* cyclic peptides can bind protein targets previously considered challenging or impossible to drug selectively using traditional small molecules (Hayashi et al., 2012; Matsunaga et al., 2016; Rentero Rebollo et al., 2016; Nawatha et al., 2019).

Understanding the structure-activity relationships of these artificial peptide-protein interactions will be critical in the development of potent cyclic peptides and their translation into the clinic. *De novo* cyclic peptides likely undergo some degree of folding upon binding (Figure 2C) (Goldbach et al., 2019). Many of the concepts and mutagenesis methods developed for natural peptide folding and binding can be recruited to understand and improve these new, drug-like peptides.

One-at-a-time non-canonical mutagenesis using SPPS has long been used to study and optimize binding peptides. An interesting recent application to cyclic peptides was a mutational scan of the bicyclic FXII618 with glycine and beta-alanine (Wilbs et al., 2016). This tested the effect of inserting CH_2_ units into the backbone and increasing the size of the macrocyclic ring(s); some of these insertions were tolerated and even improved binding affinity.

Canonical deep mutational scanning has been used to probe a cyclic peptide and its binding. A disulfide bonded peptide “meditope” was subjected to DMS, using yeast display as the selection method, to analyze multiple canonical mutations and their effect on binding to its antibody biologic target (van Rosmalen et al., 2017). However, many *de novo* cyclic peptides, by design, contain non-canonical elements, usually to enhance *in vivo* stability. For example, the flexizyme system can be used to synthesize peptides cyclized with a thioether bond, which, unlike a disufilde bond, is non-reducible (Goto et al., 2008). A powerful method to discover *de novo* binding cyclic peptides is the RaPID system, which uses this cyclization chemistry to construct enormous libraries (>10^12^) of cyclic peptides for screening using mRNA display (Yamagishi et al., 2011; Passioura and Suga, 2017).

Non-canonical scanning can be used to analyze cyclic peptides which contain non-canonical elements as part of their wild-type sequence, such RaPID *de novo* cyclic peptides. We used NCS to probe the *de novo* cyclic peptide CP2 and its binding to a histone demethylase target (Kawamura et al., 2017), testing CP2 mutations to 19 canonical and 21 diverse non-canonical amino acids (Rogers et al., 2018). Interestingly, no stabilizing mutations could be identified, suggesting the RaPID system effectively explored functional space. However, NCS did identify positions in the sequence where backbone modifications, such as N-methylation, were permissible (Figure 2D). The identification of these sites is valuable, as such modifications can improve the drug-like properties of cyclic peptides, their protease resistance and membrane permeability (Naylor et al., 2017; Walport et al., 2017).

## Discussion

Mutagenesis is a valuable tool to probe and understand the molecular interactions that govern peptide folding and binding. Historically, mutant peptides have been tested by one-at-a-time synthesis and biophysical analysis. Recently, deep mutational scanning has permitting analysis of libraries containing hundreds of thousands of mutants—a massive increase in throughput. DMS has mostly been applied to understand the folding, function, and interactions between structured proteins (Fowler and Fields, 2014). Comparatively fewer peptide-folding and -binding systems have been tested by DMS; but as more systems are tested, it will be interesting to compare their mutational behavior with those of folded protein-protein interactions. Hopefully, we can better understand the evolutionary advantage of peptide folding and binding and explain why it is so prevalent in the genomes of eukaryotic organisms (Yan et al., 2016). Importantly, DMS could help us understand the vulnerability, or not, of peptide folding and binding to pathogenic mutation (Stein et al., 2019).

How else could DMS be used to study peptide folding and binding? One possibility could be to investigate the effect of chemical or enzymatic transformations made after peptide synthesis, i.e., post-translational modifications. A “silent” encoding strategy (Tjhung et al., 2016) could be used, introducing a DNA barcode that does not affect the amino acid sequence, to follow such modifications in the DMS protocol. Another possibility is to analyze the kinetics of binding. Jalali-Yazdi et al. (2016) have showed that display techniques and NGS can be used to analyze the kinetics of binding for libraries of peptides—a protocol that could be adapted for mutational analysis and DMS. This would be a worthwhile analysis if the aim is to develop drug-candidate peptides, as kinetic parameters, namely the off-rate (residence time), can be a better predictor of *in vivo* potency than absolute binding affinity *K*_D_ (Copeland et al., 2006; Bernetti et al., 2019). Also, a kinetic approach to DMS would have the added benefit of directly measuring meaningful physical parameters, negating the need to interpret or calibrate raw enrichment ratios.

There is likely untapped mutational information in existing data sets. Many large randomized libraries have been screened and analyzed by NGS. The top recovered sequences can be aligned to guide selection of mutations to boost affinity (Huang et al., 2020). It is also possible to search for mutations of a given sequence, analyze their populations in the recovered libraries to infer structure-activity relationships (Yoshisada et al., 2017). The success of DMS shows that it is possible to extract information about relative binding affinities from screening experiments. Even though these screens were not carried out with DMS in mind, the DMS approach of calculating enrichment ratios, which corrects for uneven distributions in the library before sorting, might allow discovery of additional potency-boosting mutations.

The throughput of DMS has provided a new way to study proteins. However, there is still great value in one-at-a-time mutagenesis. First, it can generate test sets to validate a particular DMS experiment, confirming enrichment scores are truly measuring affinity, and can then be used to calibrate DMS, converting these raw scores to Δ*ΔG*/*K*_D_ values (Rogers et al., 2018). Secondly, more sophisticated biophysical analysis can be carried out when analyzing mutants one-at-a-time. Whereas, DMS is currently limited to equilibrium binding or functional measurements, one-at-a-time mutagenesis has access to the full suite of biophysical techniques, including analysis of conformation (Iesmantavicius et al., 2014) and detection of subtle differences in binding kinetics (Crabtree and Shammas, 2018). These methods allow the detailed characterization of bound and unbound states and their exchange with sparsely populated, yet critical, intermediates and transition states, to elucidate mechanisms of binding (Shammas et al., 2016; Yang et al., 2019).

Mutations to non-canonical amino acids allow for higher-resolution study of peptide structure-activity relationships, allowing subtle changes to side-chains to dissect their interactions, and modifications to the peptide backbone to probe H-bonding and secondary structure formation. Combined with the throughput of DMS, non-canonical mutagenesis can quickly and systematically assess the importance of sequence and chemical features of a binding peptide. Access to these high-resolution structure-activity maps can help optimize *de novo* cyclic peptides, either to improve potency, or to guide modifications for improved drug-like character, potentially converting promising cyclic peptide “hits” into the next-generation of chemical probes and therapeutics.

## Author Contributions

The author confirms being the sole contributor of this work and has approved it for publication.

## Conflict of Interest

The author declares that the research was conducted in the absence of any commercial or financial relationships that could be construed as a potential conflict of interest. The handling editor declared a shared affiliation, though no other collaboration, with the author at time of review.
